# Beyond unconscious bias: A practical model for improving equality and inclusion

**DOI:** 10.1016/j.clinme.2025.100309

**Published:** 2025-04-02

**Authors:** David S. Sanders, Suneil A. Raju, Anton Emmanuel

**Affiliations:** a Academic Unit of Gastroenterology, Sheffield Teaching Hospitals National Health Service Foundation Trust, Sheffield, United Kingdom; b Division of Clinical Medicine, School of Medicine and Population Health, University of Sheffield, Sheffield, United Kingdom; c GI Physiology Unit, University College London Hospital, London, United Kingdom

**Keywords:** Racism, Sexism, Equality, Diversity

It is just over 30 years since the landmark *BMJ* paper by Esmail & Everington.^1^ They developed six fictitious equivalent application CVs, which were then submitted in pairs for senior house officer (SHO) posts. The only difference between the submitted pairs was that one had an Asian name and the other an English name. For the 23 posts applied for, on 11 occasions neither candidate was shortlisted. On six occasions both candidates were shortlisted and on six occasions only the candidate with an English name was shortlisted (*p*=0.03). The Asian candidate was never shortlisted unless the English candidate was shortlisted also. Esmail & Everington were arrested and charged with making false applications. They were also threatened by the GMC with a charge of ‘behaviour that was unbecoming of the medical profession’. As a Sri Lankan-born Asian with a name of David Surendran Sanders and a 1991 graduate applying for senior house officer posts, this study did not escape my attention! I realised that in effect I was their Darwinian guinea pig. I immediately dropped my middle name and country of birth from all applications and by the time of the interview it was simply too late for this discrimination to affect me.

Racism, sexism and homophobia were all part of everyday fare for those of us growing up in the 1980s UK (Generation X). And while society has generally learned to stop using racial, sexual and disability-related slurs, somehow the inequality of progression and opportunity experienced by minoritised individuals persists. Such experiences require us to address unconscious bias and not be passive when considering issues of equality, diversity and inclusion.^2^ Such thinking is resisted by populists as ‘woke’, when actually it is simply about fairness and morality. Our children also play a profound role in developing our thinking to resist the chorus to ‘resist wokery’.

Our message is transferable to any discipline of medicine; people of colour, women and other minority groups are still under-represented at senior positions within the NHS and we suggest several methods to change this.^3^^,^^4^ Creation of fellowship programmes, mentoring and a positive workplace environment can, for example, reduce the gender gap and directly impact on unconscious biases.^5^ Fellowship programmes can be undertaken out of a training programme or after it. They allow doctors to develop expertise in an area through clinical experience and/or research, improving the employability and confidence of individuals in the process.^3^ In Sheffield, UK, we have for over 20 years provided mentorship, through the fellowship programme, for female trainees, resulting in a higher than national average female consultant body (38% versus national 22%, *p*=0.044).^6^^,^^7^ There are two other lessons learnt; first, that after mentorship comes lifetime professional allyship. Ask your female colleagues to chair and speak at meetings, value their voice, and recognise their talents by nominating them for awards. Secondly, trainees report pressures to learn in non-working hours, discriminating against those with other time pressures,^8^ like family commitments. We therefore introduced a flexible, full-payment, 4-day job plan for consultants, which has been viewed as an attractive and accommodating option.^5^

At a wider scale, introducing programmes of sponsorship at regional level resulted in 24% improved representation at executive board level compared to previous years of no notable change.^9^ These programmes were based on evidence for promoting inclusion and therefore focused on endorsing policies that reflected the company’s values and demonstrating a commitment to inclusion through supporting employees in their personal development. Additionally, 50% of trusts nationally have significant inequity, with ethnic minority staff being more likely than White peers to be referred into disciplinary processes. The Workforce Race Equality Standard report found that regions pursuing proven actions on reducing disciplinary inequality resulted in a higher number of organisations (85%) achieving racial equality.^9^, ^10^, ^11^

This framework can be applied to all forms of diversity and ensure that both ongoing conscious and unconscious biases are actively addressed.^3^^,^^4^ That is the future that Generation Z are passionately promoting and even Generation X and all in between can follow their lead.

## CRediT authorship contribution statement

**David S. Sanders:** Writing – original draft, Conceptualization. **Suneil A. Raju:** Writing – review & editing, Conceptualization. **Anton Emmanuel:** Writing – review & editing, Conceptualization.

## Declarations of competing interest

There are no declarations of interests.

Anton Emmanuel served as Editor-in-Chief of ClinMed until 2024. We hereby confirm that he was not involved in the peer-review process of this article, nor did he have access to any information regarding this process. He did not participate in the decision-making regarding the article.

## References

[1] Esmail A., Roberts C. (2013). Academic performance of ethnic minority candidates and discrimination in the MRCGP examinations between 2010 and 2012: analysis of data. BMJ.

[2] Kar P. (2024). Talk is cheap. The NHS needs anti-racist action, not empty words or virtue signalling. Br Med J.

[3] Kmietowicz Z. (2024). Little change in ethnic diversity in top roles at NHS trusts in England. BMJ.

[4] Sealy R. NHS Women on Boards 50:50 by 2020. https://www.nhsconfed.org/system/files/2021-08/NHS%20Women%20on%20Boards%20report%20%281%29.pdf (Accessed 12 March 2025).

[5] Raju S., Bowker-Howell F., Aziz I. (2024). What is the role of out of programme clinical fellowships in the era of Shape of Training? A single-centre cohort study. BMJ Open Gastroenterol.

[6] Bowker-Howell F., Kaur K., Raju S., Sanders D. (2023). Closing the gender gap in gastroenterology leadership. Lancet Gastroenterol Hepatol.

[7] RCP. Census data toolkit. https://www.rcpe.ac.uk/sites/default/files/2021_consultant_census_report.pdf.

[8] Raju S., Saunsbury E., Haddadin Y., Cowan M. (2023). Breaking the unspoken rules of UK training in gastroenterology and hepatology. Lancet Gastroenterol Hepatol.

[9] Wilkinson-Brice E, Emmanual A. NHS Workforce Race Equality Standard. https://www.england.nhs.uk/wp-content/uploads/2022/04/Workforce-Race-Equality-Standard-report-2021-.pdf.

[10] Bailey N, Martin C, Kunjachan JV. Workforce Race Equality Standard Report. https://mft.nhs.uk/app/uploads/2023/12/Workforce-Race-Equality-Standard_WRES_Report-2022-23.pdf#:∼:text=The%20WRES%20provides%20a%20framework%20for%20NHS%20organisations,monitor%20progress%20against%20nine%20indicators%20of%20workforce%20equality.

[11] England N. NHS Workforce Race Equality Standard (WRES). https://www.england.nhs.uk/long-read/workforce-race-equality-standard-2023-data-analysis-report-for-nhs-trusts/.

